# Effect of different rehabilitation training timelines to prevent shoulder dysfunction among postoperative breast cancer patients: study protocol for a randomized controlled trial

**DOI:** 10.1186/s13063-020-04954-3

**Published:** 2021-01-06

**Authors:** Yu-Wei Shao, Qing Shu, Dan Xu, Hui Teng, Gao-Song Wu, Jin-Xuan Hou, Jun Tian

**Affiliations:** 1 The Second Clinical College of Wuhan University, Wuhan, Hubei China; 2 Department of Rehabilitation Medicine, Zhongnan Hospital of Wuhan University, Wuhan, Hubei China; 3 Department of Thyroid and Breast Surgery, Zhongnan Hospital of Wuhan University, Wuhan, Hubei China

**Keywords:** Rehabilitation training, Shoulder dysfunction problems, Timeline, Breast cancer, Randomized controlled trial

## Abstract

**Introduction:**

Due to advancements in treatment, the survival of breast cancer (BC) patients has significantly improved. Improving the postoperative quality of life has become a widespread concern for patients and doctors. At present, the staged rehabilitation training program for postoperative BC patients has been recognized. However, there is not yet a consensus about the optimal time to initiate rehabilitation training. We designed this study to investigate the optimal intervention times for postoperative BC patients to begin different stages of rehabilitation.

**Design:**

This is a randomized controlled trial. Female participants with BC who are scheduled to undergo mastectomy, including unilateral total breast or breast-conserving surgery plus axillary lymph node dissection, will be enrolled in this study. The intervention includes the following: 200 participants will be allocated using a 1:1:1:1 ratio to the A, B, C, and D groups, which have four different rehabilitation timelines for four phases of rehabilitation exercises. A therapist will evaluate the patient’s overall health and then adjust the training intensity before initiating training. The assessments include upper limb mobility, grip, limb circumference, postoperative drainage volume (PDV), and pain. The training will last for 12 weeks, and patients will undergo follow-up twice within 6 weeks after discharge. Outcomes include the following: Constant-Murley Score (CMS) is the primary parameter. European Organization Research and Treatment of Cancer Quality of Life Questionnaire-BR23 (EORTC QLQ-BR23), SF-36, range of motion (ROM), strength, grip, circumference, PDV, and pain are the secondary parameters. All enrolled subjects will be assessed at 1 day, 3 days, 1 week, and 2, 3, 6, 9, 12, and 18 weeks after the surgery.

**Discussion:**

This is a randomized controlled trial to evaluate the effect of different rehabilitation training timelines to prevent shoulder dysfunction among postoperative patients with BC. If the results are confirmed, this study will establish an optimal timeline for postoperative BC rehabilitation.

**Trial registration:**

ClinicalTrials.gov NCT03658265. Registered on September 2018.

## Introduction

Breast cancer (BC) is the most commonly diagnosed malignant tumor among women worldwide. According to the statistics in America in 2018, BC alone accounted for 30% of all new cancer diagnoses in women [1]. Although China has a relatively low incidence of BC compared to America, it has increased by 20–30% in the past few decades and is growing by 3–5% annually according to the National Central Cancer Registry of China [2, 3]. Previously, the most common cause of cancer death in women age 20–59 years was BC [4]. However, early detection and surgical intervention have continued to improve, and BC survival rates now reach 90% [1].

BC treatment mainly involves surgery, along with radiotherapy and chemotherapy. The most common surgical procedures are sentinel lymph node biopsy (SLNB) and axillary lymph node dissection (ALND) for the lymph nodes [5], which are usually combined in clinical practice. SLNB is a less invasive method of evaluating nodal involvement in order to detect metastasis in a lymph node. Women with affected sentinel lymph nodes should be offered ALND [6, 7]. A meta-analysis has shown no differences between SLNB and ALND in overall survival, disease-free survival, or regional lymph node recurrence; however, it has shown significant differences in postoperative morbidity and quality of life [8]. ALND, also known as modified radical mastectomy, is indicated in patients who present with axillary metastases that are palpable or diagnosed via needle biopsy [9]. Surgery generally resects the mammary gland and the fascia of the pectoralis major and the latissimus dorsi, retaining both the pectoralis major and the pectoralis minor. During this procedure, there is a high risk of injury to the related muscles and nerves. In addition, the axillary lymph nodes and the lymphatic adipose tissue of the subscapular area are removed. After suturing and compression dressing, tissue around the armpit adheres to the muscles, forming a stiff and inelastic scar tissue, which affects upper limb mobility. Surgery combined with chemotherapy and radiotherapy may also disrupt the lymphatic reflux, which can lead to lymphedema and limit physical activity. Long-term reduced shoulder function causes chronic pain, muscle atrophy, decreased strength, decreased mobility, upper limb edema, sensory disturbance, and decreased cardiopulmonary endurance, which can severely affect patient’s emotions and quality of life [10, 11].

A meta-analysis of randomized controlled trials has shown that postoperative physical therapy has beneficial effects on BC sequelae and thus can improve shoulder function, reduce lymphedema risk, and improve the quality of life [12]. Among all kinds of therapy modalities [13–15], active exercises and stretching have been proven to be much more effective in the treatment of upper limb dysfunction than other modalities [16, 17], and accordingly, active exercises and stretching are recommended by clinical guidelines [18]. Active exercises have important physiological benefits in maintaining the blood and lymphatic flow to joints and soft tissues, which can prevent the shortening and weakness of the surrounding muscles and connective tissues that may occur following immobilization after surgery [15, 19]. At present, stretching is the widely used treatment for shoulder dysfunction after BC [16, 17]. Continuous stretching prevents contracture, tension, and adhesion of the pectoral muscles [20]. Furthermore, stretching involves the execution of movement patterns throughout the available ROM, which can improve joint mobility, enhance muscle strength, and provide a good foundation for further exercise to achieve good results [21, 22].

However, the optimal timeline for initiating postoperative rehabilitation for BC patients is not yet clear. There are many related clinical studies, and the timing in these studies ranged from postoperative 1 day to 1 year [16]. Currently, it is commonly thought that early institution of flexion and abduction exercises following axillary dissection appears to have a harmful effect on PDV and wound healing. Adequate functional ROM is attained in all patients with minimal complications when active motion exercises are delayed for up to 7 days after axillary dissection [23]. A meta-analysis has shown that researchers are more inclined to start rehabilitation exercises early. Early and delayed exercises have not been found to cause significant differences in the incidences of delayed wound healing, wound aspiration, pain, or lymphedema [16]. Therefore, we speculate that early rehabilitation is feasible for postoperative patients. We aim to investigate the effect of different timelines of multifactorial physical therapy in postoperative BC patients.

### Aim of this study

The aims of this study are as follows:

1. Observation of the optimal active exercises of the shoulder joint under full evaluation at 3 days after surgery, with respect to whether it accelerates the recovery of shoulder function and improves the quality of life of postoperative patients with BC. Evaluation of whether early-stage rehabilitation training leads to an increase in PDV, which affects shoulder function and the time of extubation in BC patients.
2. Observation of progressive resistance training (PRT) in the case of full evaluation at 3 weeks after surgery, with respect to whether it promotes the recovery of shoulder function.

## Methods and design

### Study setting and design

This is a randomized controlled clinical trial, which is currently being conducted at the Department of Rehabilitation Medicine, Zhongnan Hospital of Wuhan University. The study protocol was approved by the Medical Ethics Committee of Zhongnan Hospital of Wuhan University in September 2018. We have registered the trial content with ClinicalTrials.gov (www.clinicaltrials.gov), which is a publicly accessible primary registration authority that participated in the WHO International Clinical Trial Registry Platform (Registration time: September 2018, No. NCT03658265). The authors confirm that all ongoing and related trials are registered. All methods used in this protocol will be performed in accordance with the Standard Protocol Items: Recommendations for Interventional Trials (SPIRIT) [24].

**Fig. 1:**
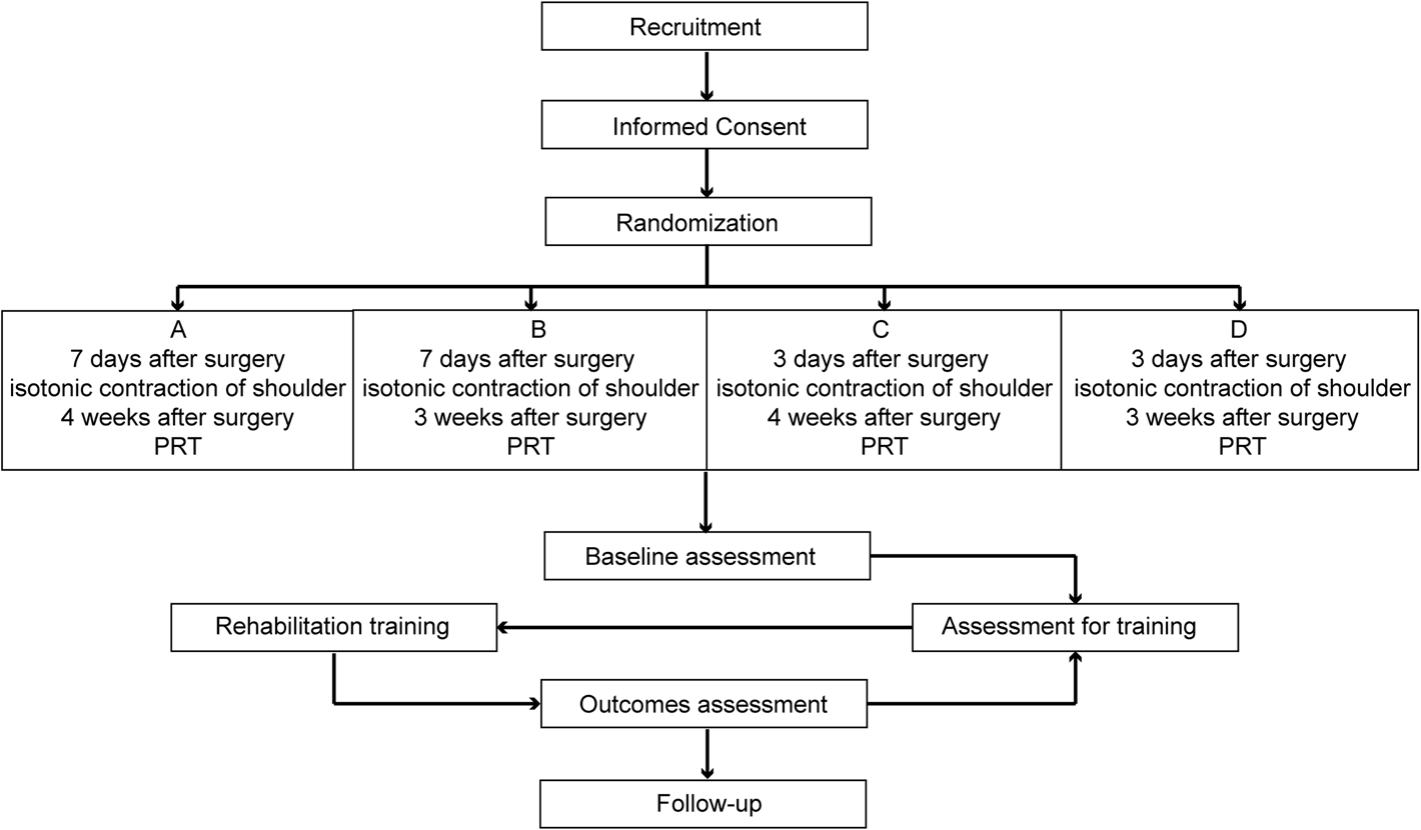
Flowchart of this study

Subjects were patients who will be recruited from the Department of Thyroid and Breast Surgery of the Zhongnan Hospital of Wuhan University from September 10, 2018, to May 31, 2020. According to the patient’s operation time sequence, we will enroll 200 patients in the trial, number them, and randomly divide them into four groups. Subjects will be asked to provide written informed consent. Subjects will perform different rehabilitation exercises at different times, and they will receive assessments from relevant physiotherapists regularly. The flowchart of this study is presented in Fig. 1.

### Participant recruitment—inclusion criteria/exclusion criteria

#### Inclusion criteria

Women will be eligible to enroll in the trial if they meet the following criteria:

(a) Between the ages of 25 and 75 years; (b) pathological diagnosis of BC; (c) patients undergoing mastectomy including unilateral total breast or breast-conserving surgery, with a positive SLNB result, for whom affected axillary lymph nodes have been removed; (d) postoperative chemotherapy or radiation therapy according to the condition; (e) no other malignant tumors within 5 years; (f) no physical therapy-related contraindications; and (g) willingness to sign the informed consent form.

#### Exclusion criteria

Patients who will meet any of the following criteria will be excluded:

(a) Comorbid tumor metastasis with other tissues and organs (liver, kidney, lung, brain, and others); (b) underlying severe heart disease, cerebrovascular disease, and mental illness; (c) patients with shoulder joint diseases and pathology before surgery; (d) negative SLNB result; and (e) unable to understand the rehabilitation training program provided by doctors and physiotherapists.

### Concealed randomization and blinding

Prior to the study, a computer-generated random sequence will be generated by an external researcher who is not otherwise affiliated with the study, and the sequence will be concealed in opaque envelopes. Group assignment will be disclosed to the executive doctor by telephone after study inclusion and participation in the familiarization period. Participants will be allocated using a 1:1:1:1 ratio to the A, B, C, and D groups.

The blinding to division results will be maintained by the study designer until the end of the study to ensure concealment of group allocation, and thus, the outcome assessor and the data analyst will also be blinded. The subjects and the therapists will be instructed not to disclose the allocation to the outcome assessor, and accidental unblinding will be documented.

### Assessment for training

After providing informed consent, participants will be asked standardized questions about their demographic characteristics and medical history. Clinical data will be extracted from the medical records regarding primary tumor, time of primary diagnosis, surgical operation, physical examination, laboratory testing, and imaging examination. To ensure the safety of the training, a therapist will evaluate the patient’s overall health, including physical function, physical activity, mental status, and PDV. Next, the training intensity will be adjusted before patients begin the training. The assessments will include upper limb mobility, grip, limb circumference, PDV, and pain.

Upper limb mobility is mainly reflected in both ROM and muscle strength. A medical protractor is usually used to assess the range of shoulder motion and follow standardized measurement principles to reduce compensatory motion. Passive flexion and abduction of the shoulder will be assessed in a supine position with gravity as a standard resistance. Active flexion and abduction will be assessed in a sitting position. Manual muscle test (MMT) and dynamometer are generally used to measure the maximum strength of the shoulder muscles. Strength evaluation will require the patients to take the supine position in the early stage and then gradually change to the sitting position in the late stage. The arms will be fixed at 90° to assess flexion and abduction muscle strength. The patient will be asked to perform three maximum voluntary contractions in each direction, recording the highest data from three attempts. Each arm will be measured separately. The most common method to determine whether the upper extremity has edema is the measurement of upper limb circumference. Using the antecubital fossa and palm as location points, the circumferences of the bilateral arms in a specific position will be measured, and the differences will be compared between the two sides. If the difference between the two sides is greater than 2 cm at the same site, it will indicate edema. PDV and pain will be assessed through consultation. PDV will be recorded by the nurse and will be uploaded to the hospital work system regularly. The therapist will ask the nurse about PDV and pain or directly search the system. Daily pain or pain at the end of the active/passive range of each exercise will be measured clinically using a standardized 10-cm visual analog scale (VAS). VAS is widely used clinically to assess postoperative pain and to monitor the effectiveness of treatment. VAS is based on self-reporting via a unidimensional scale that aims to represent subjective pain intensity [25, 26].

The six measurements will be tested at postoperative 1 day, 3 days, and 1 week, after which the patients will visit the hospital for evaluation once every other week. The ROM, muscle strength, and grip strength results will be used as criteria for maximum exercise intensity settings, and pain, PDV, and limb circumference will be used as indicators to assess whether exercise intensity is appropriate. Exercise intensity will set based on the current test results. If the evaluation index deteriorates, the intensity should be adjusted appropriately.

### Training sessions

First, all patients will undergo preoperative assessment and rehabilitation education, including investigation of the patient’s basic information, medical history, and therapeutic schedule, and they will be taught how to undergo rehabilitation. Patients will be taught according to the special BC Rehabilitation Mission Manual to strengthen their understanding regarding the importance of postoperative rehabilitation of BC and to increase their compliance.

The exercise program will be divided into four phases. Phase 1 will consist of the following four movements: (1) The isometric handgrip exercise will be adapted for 2 min of 5-s contraction and 5-s rest-pause intermittently per set, 3 sets per day. (2) The isotonic contraction exercises of the wrist and elbow will not be loaded. Full range of joint motion will be performed, 10–15 times per set in each direction, 3 sets per day. (3) Isometric contraction of the shoulder joint will be performed in both the flexion and abduction directions. Muscles will be allowed to contract for 10 s and rest for 5 s, 10–15 times per set in each direction, 3 sets per day.

Phase 2 will be the isotonic contraction of the shoulder joint in both flexion and abduction. According to the pre-training assessment results, researchers will adjust the appropriate ROM for patients with gravity as the load, 10–15 times per set in each direction, 3 sets per day.

Phase 3 will involve active stretching of the shoulder flexion and abduction. The patient will be asked to face or side face the wall, place her hand on the wall, and slowly crawl her fingers upward. When the patient feels that the armpit has a slight pulling or pain, she will be asked to stop moving her fingers, stretch the shoulder joint for 20–30 s, and then slowly move the arm away from the wall. The same movement will be repeated 10–15 times per set, 3 sets per day.

Phase 4 will involve PRT of shoulder flexion and abduction. Based on the ROM and muscle strength results of the pre-training assessment, the researchers will adjust the appropriate resistance to allow the patients to lift repetitively for 8–15 times per set, 3 sets per day. Providing resistance can be achieved through equipment, such as elastic bands and barbells.

All subjects need to perform the routine exercises of phases 1 and 3, regardless of group assignment. According to the different group assignments, the patients will perform the exercises of phases 2 and 4 at different times. Relevant concomitant care is permitted for all patients.

### Training timelines

The patients will be divided into four groups: A, B, C, and D. All patients will start the rehabilitation training of phases 1 and 3 separately on the first day and the second week after surgery. The training timings of phases 2 and 4 are slightly different in different groups. Group A will start phase 2 at 1 week after surgery and phase 4 at the fourth week after surgery. Group B will also start phase 2 at 1 week after surgery, but they will start phase 4 in the third week after surgery. Group C will advance the start of phase 2 to the third day after surgery, but they will not start phase 4 until the fourth week after surgery. Group D will not only advance the start of phase 2 to the third day after surgery, but they will also initiate phase 4 to the third week after surgery, 1 week ahead of schedule.

During the trial, patients will stay in the hospital or return to the hospital regularly for chemotherapy, to ensure that they can be directly instructed. After patients are fully discharged, the training meeting will be instructed and completed in the form of a home exercise, program lasting for 6 weeks, 3–5 sets per week. A video of each patient’s exercise session should be sent to the therapist for examination.

### Outcomes

#### Primary parameter

CMS is a method that records individual parameters and provides an overall clinical functional assessment of the shoulder, and it is sufficiently sensitive to reveal even small changes in shoulder function [27, 28]. The score consists of the following four domains: pain (1 item), activities of daily living (ADL; 3 items for activity level, i.e., work, sports, sleep; 1 item for hand positioning, i.e., rotation), mobility (4 items: forward and lateral abduction/elevation, external and internal rotation), and strength (1 item) [29]. Pain and the three items for the activity level of ADL will be obtained from the patient, which means that patients will be self-assessed, while all other items will be assessed by the physiotherapist. The method of assessment will consist of clinical examination plus patient interview. The complete assessment only requires 5–7 min to perform [30]. All items are easy to understand and not emotionally sensitive. The respondent burden is minimal. The assessment of retest reliability using the intraclass correlation coefficient is 0.80–0.96, which indicates that the items in each scale are highly correlated with one another compared with items of another scale [31].

#### Secondary parameters

Quality of life (QOL) is a multidimensional, subjective construct that reflects physical, mental, and social health domains of patients [32]. The European Organization Research and Treatment of Cancer (EORTC) QLQ-BR23 questionnaire is one of the most available tools for assessing aspects of QOL for BC patients [33]. It contains 23 items divided into two functional scales, including body image and sexuality, and three symptom scales, including arm symptoms, breast symptoms, and systemic therapy side effects. This questionnaire has been found to have a high internal consistency for 4 out of 5 scales and well-known group discriminative ability [34]. The validity of the EORTC QLQ-BR23 has been internationally scrutinized and explored in many countries, which has ultimately confirmed the questionnaire’s validity in BC [35].

The SF-36 is a general brief self-report questionnaire that assesses health-related QOL. The SF-36 contains 36 items that measure eight multi-item variables, including physical functioning, social functioning, role limitations due to physical problems, role limitations due to emotional problems, mental health, energy and vitality, bodily pain, and general perception of health, and there is a single-item scale on health transition. The score of each item ranges from 0 to 100. For each variable, the item scores are calculated, and a higher score indicates a better health status [36]. The internal consistency of the variables in the SF-36 is assessed with Cronbach’s α statistic and test-retest reliability [37]. Studies have shown that the SF-36 is acceptable for patients, and it has a high level of internal validity and good test-retest properties [38].

The assessments of shoulder function and quality of life will be evaluated at 1 week, 3 weeks, and 9 weeks after surgery.

In addition, joint mobility, muscle strength, grip, circumference, PDV, and pain, the parameters of exercise intensity in the pre-assessment, will also be evaluated as outcomes. Assessment methods and principles are the same as those for pre-assessments.

Shoulder ROM and strength will be assessed at postoperative 3 days and 1, 2, 3, 6, and 9 weeks. Grip will be evaluated at postoperative 1 day, 3 days, and 1, 2, 3, and 6 weeks, and upper limb circumference will be assessed at postoperative 1 day and 3, 12, and 18 weeks. PDV, pain, and skin will be evaluated by interview and palpation at postoperative 1 day, 3 days, 1 week, and 2 weeks.

CMS, EORTC QLQ-BR23, and SF-36 will be required to be completed during the follow-up sessions at 12 and 18 weeks after surgery. During the follow-up sessions, information related to clinical testing, laboratory testing, and adverse events will be collected and assessed. Patients will be followed up in the ward during chemotherapy, and it is supplemented by telephone or WeChat.

**Fig. 2:**
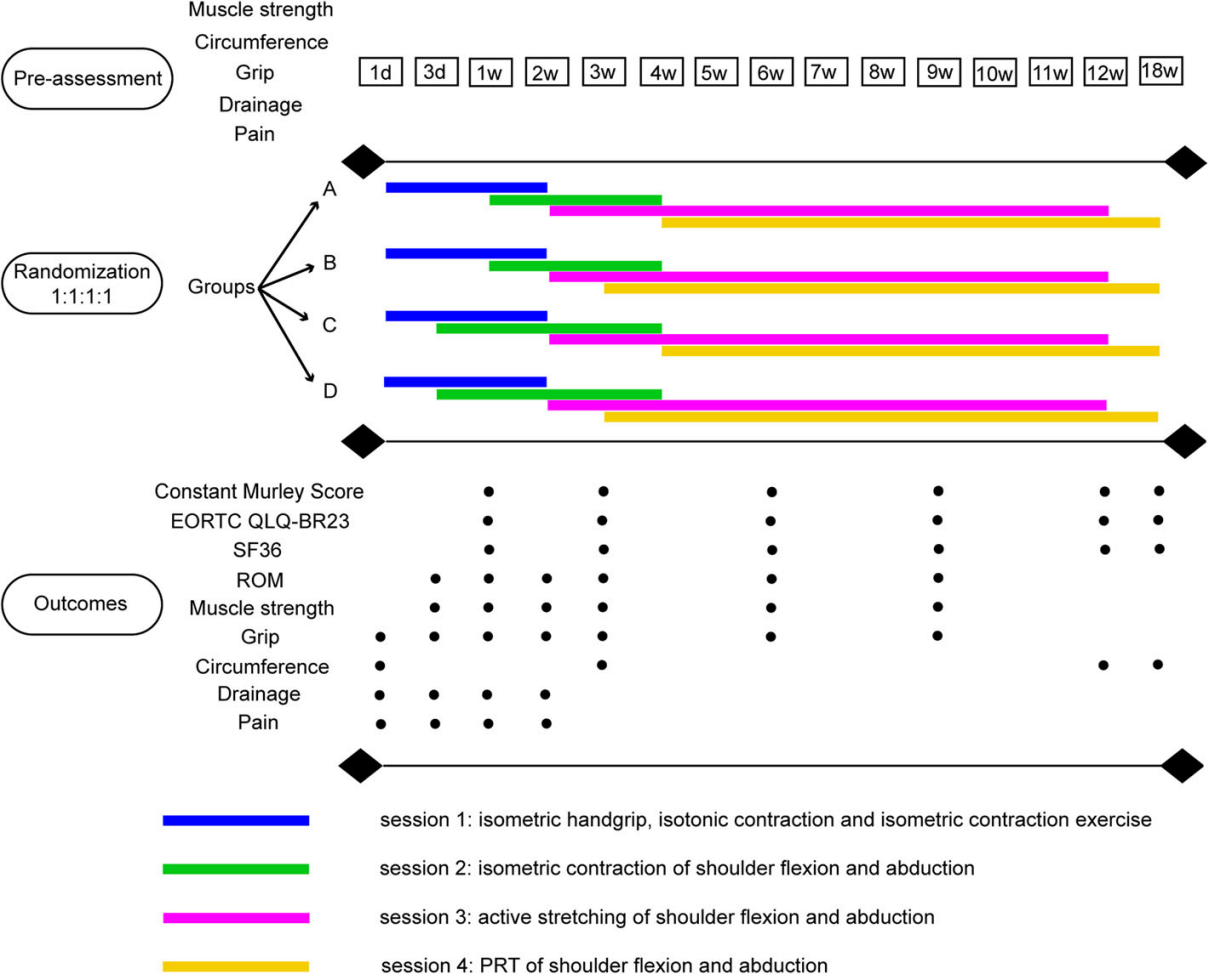
The study design

Figure 2 shows a diagram of the study design.

### Data analysis

#### Sample size

By estimating the sample size of the k-group sample mean comparison in a completely random design, the improvement scores for the CMS of the four groups of patients at 84 days after treatment were 15, 16, 18, and 19, and the standard deviations were 2, 2.5, 2.5, and 3, respectively. The assumption was that *α* = 0.05 and 1-*β* = 0.9. After calculation using PASS software (11.0), a total of 164 patients were required to be treated, and a 20% loss rate was calculated. Each group required 50 patients [39].

The calculation formula was as follows:

$$ n=\frac{\psi^2\left[{\sum}_{i=1}^k\frac{S_i^2}{k}\right]}{\left[{\sum}_{i=1}^k\frac{{\left(\overline{X_i}-\overline{X}\right)}^2}{\left(k-1\right)}\right]}. $$

### Statistical analyses

Analyses will be performed using SPSS statistical software (version 20.0) by a statistician in the Department of Rehabilitation Medicine, Zhongnan Hospital of Wuhan University. Data will be analyzed according to the “intention-to-treat” (ITT) principle. A two-sided significance level of 5% will be used for all analyses. If the experimental data satisfies the normal distribution and the homogeneity of the variance, one-way analysis of variance (ANOVA) will be performed for comparison among the 4 groups with respect to the CMS, EORTC QLQ-BR23, SF-36, ROM, strength, grip, circumference, PDV, and pain. If there is an overall difference, the Tukey method for multiple comparisons will be used to compare the differences between groups at each time point. The results of repeated measurement ANOVA testing at each time point in each group will be analyzed by repeated measures to observe the recovery of patients at different times after surgery. For data with irregular variance, variable transformation can be used after the Kruskal-Wallis *H* test.

### Data monitoring and ethical consideration

All personal data will be treated in accordance with the existing rules and regulations. A unique trial identification number will be used for searching subjects. Clinical and patient forms will be checked for completeness and congruity before data entry into the database. Data will undergo additional checks monthly by the supervisor from the Clinical Trial Center of Zhongnan Hospital of Wuhan University to ensure consistency between the data submitted and the original paper forms.

On the consent form, participants will be asked if they agree to the use of their data should they choose to withdraw from the trial. Participants will also be asked for permission for the researchers to share relevant data. A safety reporting protocol has been developed for related and unexpected serious adverse events and directly attributable adverse events (AEs). Any AE that occurs while undertaking exercises, either during an appointment or while exercising unsupervised at home, requires a report to the therapist. The supervisor has the right to terminate the trial when serious AEs occur. The researchers found through preliminary experiments that the anticipated problem of harm to subjects, such as non-healing wounds, was not observed. The trial Chief Investigator determines whether AEs require reporting to the Clinical Trial Center of Zhongnan Hospital of Wuhan University, in accordance with the full safety reporting protocol.

## Discussion

Functional axillary dissection has been widely performed in breast surgery at the Department of Thyroid and Breast Surgery of the Zhongnan Hospital of Wuhan University [40]. Surgery for BC has improved and has also been supplemented with endocrine therapy, targeted therapy, and radiation therapy to maintain high disease-free survival while reducing surgical trauma. Based on the previous research results [12, 17], this study aimed to design a more effective exercise program for postoperative patients undergoing rehabilitation. Although relevant guidelines already exist, they have not been updated regularly. Some researchers have conducted a prospective study on the timing of rehabilitation exercise, and they found that early rehabilitation exercise had no significant effect on wound healing and did not increase the risk of lymphedema [16]. We wondered whether the optimal intervention times of the rehabilitation methods recommended in the guidelines could be advanced as surgical trauma is reduced. Therefore, we set up different rehabilitation timelines combined with exercise to verify the feasibility of the earlier rehabilitation exercise process.

In the Clinical Practice Guidelines for Breast Cancer Rehabilitation, it is recommended that postoperative physical therapy should begin the first day following surgery, gentle ROM exercise should be initiated in the first week after surgery, and the resistive exercises can be started with light weights within 4–6 weeks after surgery. Active stretching exercises can be initiated 1 week after surgery, or when the drain has been removed, and they should be continued for 6 to 8 weeks or until full ROM is achieved in the affected upper extremity [18]. According to the guidelines, we designed a timeline initiating active shoulder exercises 1 week after surgery and PRT 4 weeks after surgery as the control condition for this study.

Thus far, many researchers have considered that rehabilitation exercises should be initiated as early as possible, and therefore, researchers have advanced the rehabilitation time to 1–3 days postoperative [41–43]. However, these studies from countries around the world have assessed shoulder joint activity in the very early rehabilitation period without considering the recovery process of the upper extremity. Some of these studies have unclear restrictions on shoulder activity, and some do not even limit activity, which must be improved. Therefore, we combined with the existing research and the guidelines to determine that patients should begin an activity on the first day after surgery, and designed isometric and isotonic exercises for adjacent joints. The isometric exercise is a type of post-isometric relaxation exercise, while the isotonic exercise is rhythmic relaxation and contraction of muscles, both of which act as a muscle pump to promote the circulation of lymph fluid and blood and maintain muscle flexibility and elasticity [44]. We have designed the optimal intensity to reduce the impact of muscle fatigue as much as possible, and we have also ensured the enthusiasm of patients’ activities [45]. Based on several studies, we have taken the above activities as a warm-up and have chosen the third day after surgery as the time to initiate shoulder joint activities to ensure the safety and effectiveness of the exercise. ROM and intensity of shoulder exercises will be strictly controlled by the physiotherapists.

PRT is a modality of active exercise that can potentially address many side effects of BC, improving both shoulder function and quality of life [46]. Considering that PRT is a high-intensity upper limb training modality, a meta-analysis indicated that PRT is sufficiently reliable for reducing the risk of lymphedema and improving shoulder function [47]. We will follow the step-by-step principles in the guidelines to design the optimal resistance. Although the recommendations for the PRT clinical guidelines indicate that it should be initiated from the fourth week after surgery, majority of the studies have suggested that the participants performing the PRT intervention should concurrently receive chemotherapy, which is usually initiated at 2–3 weeks after surgery [48, 49]. As chemotherapy can easily lead to osteoporosis, muscle weakness, and physical pain [50], we believe that progressive resistance training should be started early, because resistance training can improve the body metabolism and enhance physical fitness [51]. Therefore, we decided to initiate the PRT in the third week after surgery to observe its effect on shoulder function recovery. Our protocol design can only be as comprehensive as possible, but too many influencing factors can lead to many shortcomings in research. Therefore, further research is required to advance the general knowledge in this area to improve the prescription of active exercises as a component of mainstream medical practice, and define the optimal rehabilitation training timing for BC patients.

## Trial status

At the time of manuscript submission, enrollment into the study is still ongoing. Subjects are patients who were recruited from September 10, 2018, to May 31, 2020. Protocol version 4.0, October 28, 2020.

## Data Availability

The datasets analyzed during the current study are available from the corresponding author upon reasonable request. Any data required to support the protocol can be supplied on request.

## Abbreviations

BC: Breast cancer
PDV: Postoperative drainage volume
CMS: Constant-Murley Score
SLNB: Sentinel lymph node biopsy
ALND: Axillary lymph node dissection
ROM: Range of motion
PRT: Progressive resistance training
SPIRIT: Standard Protocol Items: Recommendations for Interventional Trials
MMT: Manual muscle test
VAS: Visual analog scale
ADL: Activities of daily living
QOL: Quality of life
EORTC QLQ-BR23: European Organization Research and Treatment of Cancer Quality of Life Questionnaire-BR23
ANOVA: Analysis of variance
